# Application of iontophoresis in ophthalmic practice: an innovative strategy to deliver drugs into the eye

**DOI:** 10.1080/10717544.2023.2165736

**Published:** 2023-01-11

**Authors:** Dong Wei, Ning Pu, Si-Yu Li, Yan-Ge Wang, Ye Tao

**Affiliations:** a Henan Eye Institute, Henan Eye Hospital, People’s Hospital of Zhengzhou University, Henan University School of Medicine, Henan Provincial People’s Hospital, Zhengzhou, China; b College of Medicine, Zhengzhou University, Zhengzhou, China

**Keywords:** Drug delivery, ocular, electric current, iontophoresis

## Abstract

Delivery of drugs to special locations of ocular lesions, while minimizing systemic and local toxic effects, is recognized as a critical challenge in the ophthalmic practice. The special anatomy and physiology barriers within the eyeball entail effective drug delivery systems. Emerging attempts in drug delivery has led to developments in ocular iontophoresis, which acts as a noninvasive technology to enhance drug penetration using a small electric current. This technique offers greater flexibility to deliver desired drug dose in a controlled and tolerable manner. In previous studies, this technique has been testified to deliver antibiotics, corticoid, proteins and other gene drugs into the eye with the potency of treating or alleviating diverse ophthalmological diseases including uveitis, cataract, retinoblastoma, herpes simplex and cytomegalovirus retinitis (CMVR), etc. In this review, we will introduce the recent developments in iontophoresis device. We also summarize the latest progress in coulomb controlled iontophoresis (CCI), hydrogel ionic circuit (HIC) and EyeGate II delivery system (EGDS), as well as overview the potential toxicity of iontophoresis. We will discuss these factors that affect the efficacy of iontophoresis experiments, and focus on the latest progress in its clinical application in the treatment of eye diseases.

## 1. Introduction

Eye is a special organ with immune privileges and consolidate architectures that is anatomically divided into two segments (Myles et al., 2005). The anterior of the eye, specifically the cornea, is hypovascular and transparent for unobstructed vision and is exposed to the environment (Cabrera et al., 2019). In addition, a static barrier of the corneal consists of corneal epithelium, epithelial tight junctions, corneal stroma, corneal endothelium and blood aqueous. The barrier function of the cornea leads to less drug absorption and a very low permeability to the penetration of foreign molecules (Mofidfar et al., 2021). Furthermore, if the drug narrowly crosses the corneal barrier, it will still be blocked by the iris-lens barrier when it drives to posterior of the ocular. The posterior segment of the eye is vascularized, fragile, and difficult to perform noninvasive therapy (Cabrera et al., 2019). Many irreversible eye diseases occurred within the posterior segment of the eye. Although most of these diseases cannot be cured now, we can still use drugs to slow down the development of the disease. If the drug is delivered from the sclera to bypass the cornea, the static barrier within sclera is difficult to handle. This static barrier includes the conjunctiva, sclera, choroid, Bruch’s membrane, and retina (Li & Hao, 2018). In addition to the above static barrier troubles, blood-ocular barrier is also a thorny problem during drug administration. The blood-ocular barrier is made up of the blood–aqueous barrier (BAB) and the blood–retinal barrier (BRB). In the two blood–ocular barriers, the BAB regulates the exchange between the circulating blood and the aqueous humor, while the BRB regulates the exchange between the circulating blood and the neural retina (Tomi & Hosoya, 2010; Thrimawithana et al., 2011).

### 1.1. Current restrictions in ocular drug delivery

Delivery of drugs to the eyeball, especially the posterior segment, is a challenging problem due to several anatomic barriers and lacrimal drainage, as well as the long diffusion distance (Varela-Fernández et al., 2020). For instance, BRB poses pharmaceutical challenges by limiting the perfusion of the drug from the highly vascular choroid into the retina and vitreous (Kubo et al., 2018). To achieve a therapeutic concentration in the vitreous, the drugs have to pass through this thorny barrier. However, the β-lactams (cefazolin) and aminoglycosides (amikacin), which are regularly used to treat endophthalmitis, cannot cross BRB efficiently to reach adequate vitreous concentrations via systemic delivery (Yannuzzi et al., 2017). Besides, extensive drug dilution in the blood, low cardiac output to the eye, systemic drug metabolism by the liver and systemic drug clearance by the kidney, are also resulting in only a small quantity of the drug typically reaching the vitreous humor (Yavuz & Kompella, 2017). For this reason, systemic delivery approach usually requires high doses and frequent dosing that would cause side effects on other unwanted organs of the body. Intravitreal injections can directly deliver drugs to the eyeball and its administration rate reaches 100%, but these invasive procedures of injections might cause a series of adverse events including hemorrhage, retinal detachment, endophthalmitis, sustained ocular hypertension, lens injury, development or progression of cataract, or hypotony (Doshi et al., 2011; Lashay et al., 2020; Mautone et al., 2021). Topical application is a safe and convenient approach to deliver ocular drugs on the eye surface. It is mostly in the form of eye drops, employed to treat anterior segment diseases (Gaudana et al., 2010). In fact, Even if a lower molecular weight can reach the posterior segments of eye through corneal and noncorneal pathways, it is still very difficult to treat the posterior segments of eye diseases by topical administration due to the obstruction of the various eye barriers. For instance, these factors like lacrimation, aqueous humor circulation, and biological barriers would restrain the penetration of topically applied drugs to attain therapeutic concentrations in the lesions of posterior eyeball. Due to the rapid clearance of drugs from the ocular tissue, developments of technologies that prolong drug residence and enhance ocular accessibility remain key attributes for medications. The periocular route not only reduces various dangerous sequelae after intravitreal injection, but also bypasses the corneal barrier and the iris-lens barrier, directly entering the posterior of the eye from the sclera which has larger area and better drug absorption compared with cornea. However, in the face of acute diseases in the posterior segment of the eye, its therapeutic effect is far less than that of vitreous injection (Raghava et al., 2004). If the drug transport rate of the above methods is high, it will face greater side effects. On the other hand, if the side effects are small, the drug transport rate is not satisfactory. Therefore, safe improvement in transport efficiency acts as a critical precondition to the treatment of the posterior segment of the eye disease.

### 1.2. Mechanism of iontophoresis

Iontophoresis is a noninvasive approach which drives the penetration of pharmacological molecules across anatomic barriers using a small electric current. This technique has been used in a broad spectrum of pharmacological experiments and clinical conditions (Pandey et al., 2019; Vinciguerra et al., 2021). Generally, the iontophoresis uses two types of voltage supply-direct current and alternating current. The most commonly used method is direct current iontophoresis (Karpiński, 2018). The crux of iontophoresis is to take advantage of electrical field to prompt the drugs penetration through media, cell membranes, or tissues, respectively. The amount of delivered therapeutic molecules is about 10–2000 times greater than conventional forms. This method has been used in dentistry, ophthalmology, otorhinolaryngology, and dermatology (Karpiński, 2018). The central tenet of iontophoresis is built on the basic electrical principle in which oppositely charged ions attract and same charged ions repel (Eljarrat-Binstock & Domb, 2006). In ophthalmic practice, iontophoresis enhances drug penetration across biological membranes via the following mechanisms: direct-field effect, electroosmosis and electropermeabilization.

The direct-field effect, also called the Nernst-Planck effect, is based on the principle of ion movement caused by an applied electrical potential gradient. The ionized substances are attracted by direct-field effect to anode or the cathode depend on the charge. The direct-field effect, is the largest contributor to flux enhancement for small ions, but not the only one. Electroosmosis, also called the Electroosmotic flow, is the bulk fluid flow which occurs when a voltage difference is imposed across a charge membrane (Eljarrat-Binstock & Domb, 2006). The motion of the solvent can enhance the transport of ionic and neutral drugs. Electroosmosis is a dominant mechanism for the enhanced transport of large monovalent ionic during iontophoresis. Electropermeabilization is the alteration of a tissue barrier under the influence of an electric field that can increase the permeability of the tissue during and after iontophoresis (Li & Hao, 2018). The porosity of a membrane and the properties of the transport pathways in the membrane can be altered by the electric field. For neutral molecule, the electroosmotic flow is the major mechanism. Siva Ram Kiran Vaka et al found that the transport enhancement by iontophoresis was predominantly caused by the electrophoresis and/or electro-osmosis (Vaka et al., 2008).

## 2. Routes of iontophoresis administration

**Figure 1.**
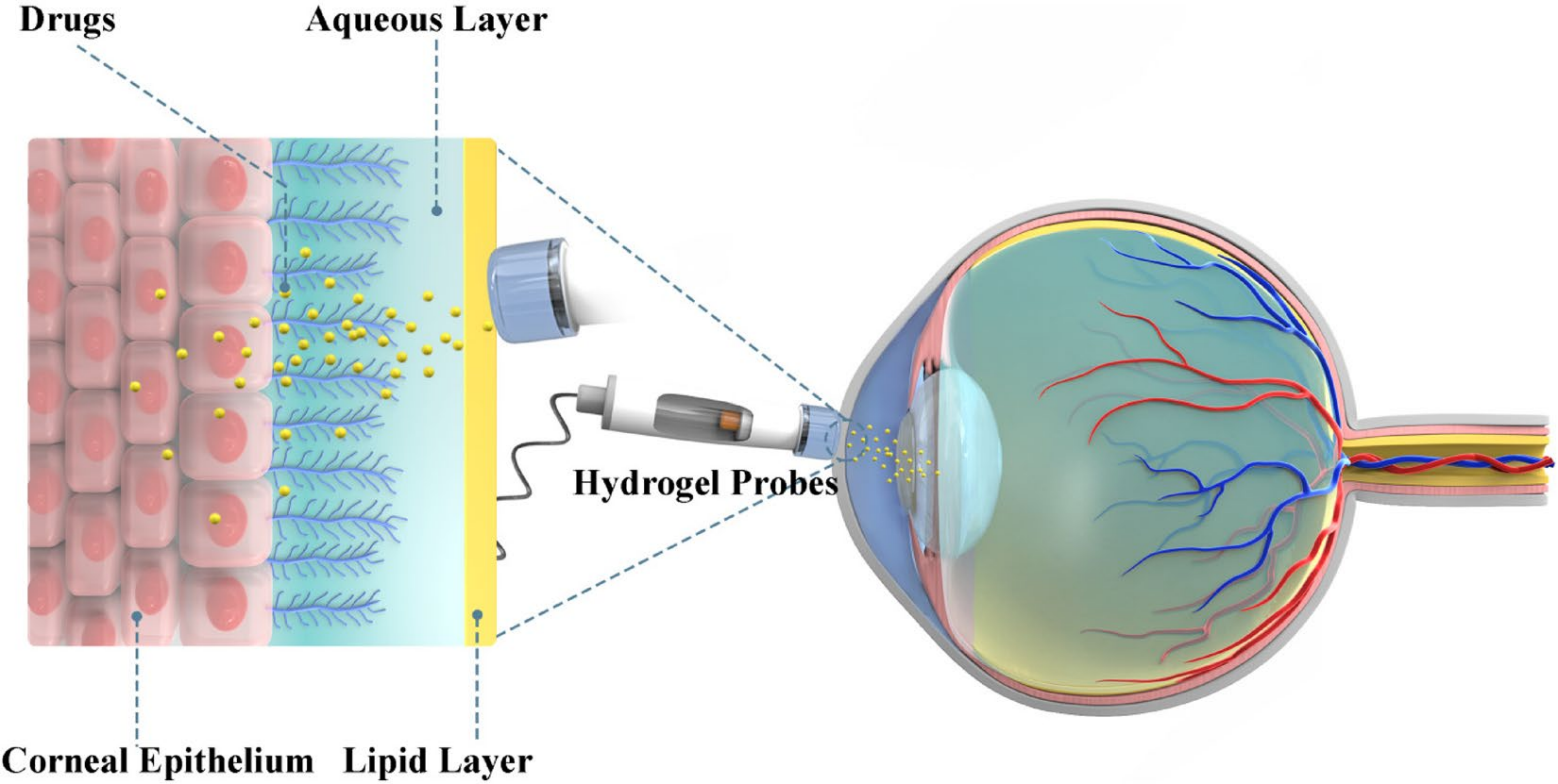
Schematic diagram of drug release and penetration by transcorneal iontophoresis.

**Figure 2.**
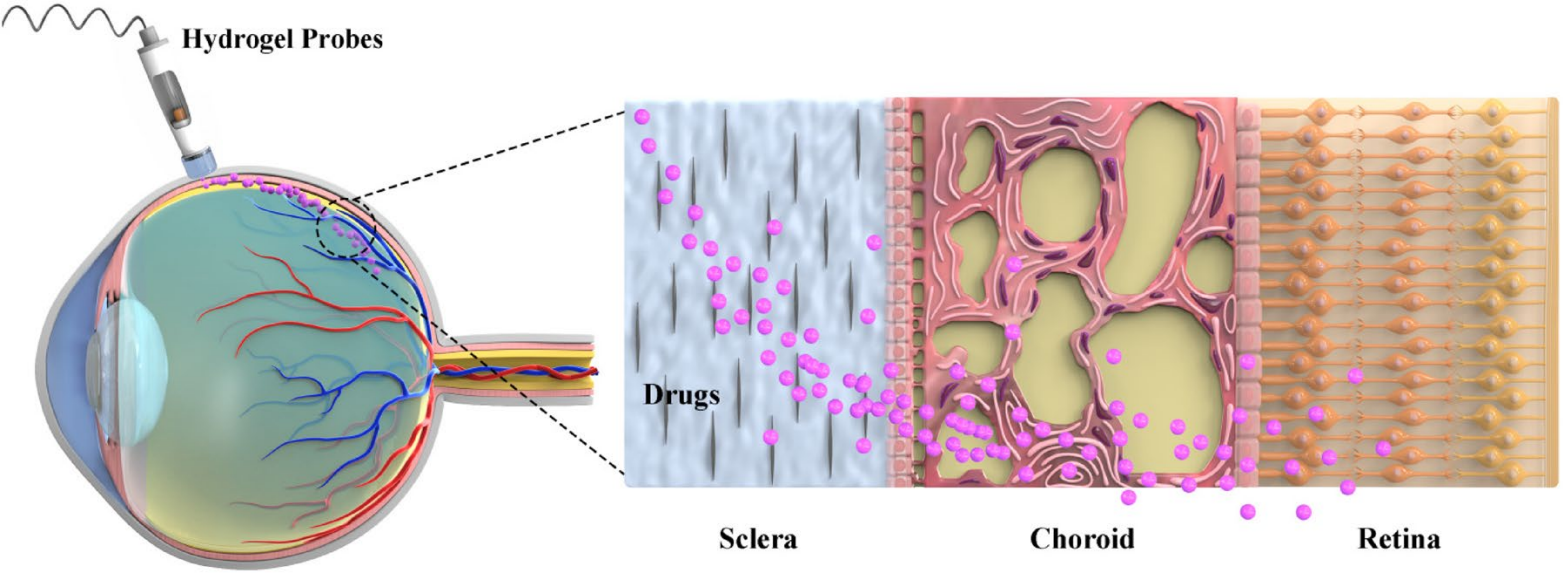
Schematic diagram of drug release and penetration by transscleral iontophoresis.

In ophthalmic practice, iontophoresis has been used to drive antibiotics to produce high concentrations of pharmacological molecules in the vitreous humor, and its safety and efficiency have been testified by numerous animal experiments and clinical trials (Table 1) (Güngör et al., 2010; Perez et al., 2020). Typically, iontophoresis can be divided into transcorneal and transscleral iontophoresis according to the application site. Transcorneal iontophoresis has been developed to treat anterior segment diseases by delivering antibiotics such as gentamicin, tobramycin, ciprofloxacin and vancomycin (Eljarrat-Binstock et al., 2010). Cornea is composed of collagen structure. In particular, the stratified, squamous and non-keratinized corneal epithelium is the most critical barrier to penetration with a 10^−7^–10^−5 ^cm^−1^ drug permeability rate because of the fact that tight junctions impair the permeation of low lipophilic molecules (Varela-Fernández et al., 2020). During iontophoresis, adjusting the current density to 6.25 mA/cm^2^ will increase the drug load to 120 ng/mg in the cornea. The accumulation of drug in the cornea can create an in situ drug depot that enabled a slow sustained release of drug into the aqueous humor and thus eliciting a prolonged therapeutic effect (Gratieri et al., 2017). Normally, transcorneal iontophoresis has achieved some good results in delivering high and sustained drug concentration to the anterior segment (Figure 1). However, due to the lens barrier, the introduced drug cannot reach the posterior segment of eyeball (Eljarrat-Binstock & Domb, 2006; Eljarrat-Binstock et al., 2010). In this context, transscleral iontophoresis is developed to eyeball meet these requirements (Figure 2). Numerous small molecular antibiotics such as amikacin, cefazolin, Gentamicin, ticarcillin have been delivered thorough this mode (Myles et al., 2005). Sclera higher degree of hydration, lower number of cells, and larger surface area than the cornea (about 17 cm^2^ versus 1.3 cm^2^). Therefore, it is permeable to large molecular weight compounds (Del Amo & Urtti, 2008). In phakic animals, the lens-iris diaphragm limits the penetration of a topically applied drug to the posterior tissues such as vitreous and retina. Transscleral iontophoresis can overcome this barrier and deliver drugs into the vitreous and retina through the choroid (Eljarrat-Binstock & Domb, 2006). Using a solid hydrogel probe, the researchers sprayed gentamicin sulfate through the sclera to deliver the drug to the posterior segment during low current iontophoresis. The results show that this device can be used to obtain high concentrations of drugs to the posterior segments of the eye using a short transscleral iontophoresis with low current. This convenient strategy can be used to treat various posterior infections of the eye, i.e. posterior uveitis, scleritis or endophthalmitis. In previous clinical practice, these infections can only be treated with invasive intravitreous injections of antibiotics, which are associated with severe complications and pain (Eljarrat-Binstock et al., 2004).

**Table 1.** Novel experimental studies on the application of iontophoresis to ocular lesions.

Type of ocular diseases	Route	Drug	Experimental animals	Disease models	Reference
DED	Transcorneal	Iodide, Dex-Phos	Human	Human diseases	(Horwath-Winter et al., 2005; Patane et al., 2011)
Pathologic myopia	Transscleral	Riboflavin, UVA SXL (i-ASXL)	Rabbits	Relatively stable myopia by visual deprivation	(Rong et al., 2017)
Uveitis	Transcorneal or Transscleral	Dex-Phos, ODNs, L-NAME	Rat, Human	EIU, Human diseases	(Behar-Cohen et al., 1997; 1998; Voigt, de Kozak, et al., 2002; Cohen et al., 2012)
Herpes simplex infections	Transcorneal or Transscleral	ACV-X prodrugs	Rabbits	HSV-1 McKrae strain induced eye inflammation	(Chen & Kalia, 2018)
Noninfectious scleritis	Transscleral	Dex-Phos	Human	Human diseases	(O’Neil et al., 2018)
DR	Transscleral	Insulin, bevacizumab	Rat	STZ induced diabetic, human diseases	(Koevary et al., 2002; Pescina et al., 2010)
Rb	Transscleral	Carboplatin	Mouse	The LH_BETA_T_AG_ model of Rb	(Hayden et al., 2006)
CMVR	Transscleral	Ganciclovir, foscarnet	Human	Human diseases	(Lam et al., 1994; Port et al., 2017; Chaudhry & Fung, 2021)
CNV	Transscleral	Bevacizumab	Rabbits	Adeno-associated virus encoding human VEGF-165 induced CNV	(Molokhia et al., 2020)

Abbreviations: DED, dry eye disease; DR, diabetic retinopathy; Rb, retinoblastoma; CMVR, cytomegalovirus retinitis; CNV, choroidal neovascularization; Dex-Phos(EGP-437), dexamethasone phosphate; ODNs, anti-sense oligonucleotides; UVA, ultraviolet A; SXL, scleral collagen cross-linking; i-ASXL = iontophoresis-assisted accelerated riboflavin/UVA SXL; L-NAME, n′-nitro-L-arginine methyl ester; ACV-X, acyclovir (X = Arg, Gly and Trp); EIU, endotoxin-induced-uveitis; HSV, herpes simplex virus; STZ, streptozotocin; VEGF, vascular endothelial growth factor.

## 3. The iontophoretic device

**Figure 3.**
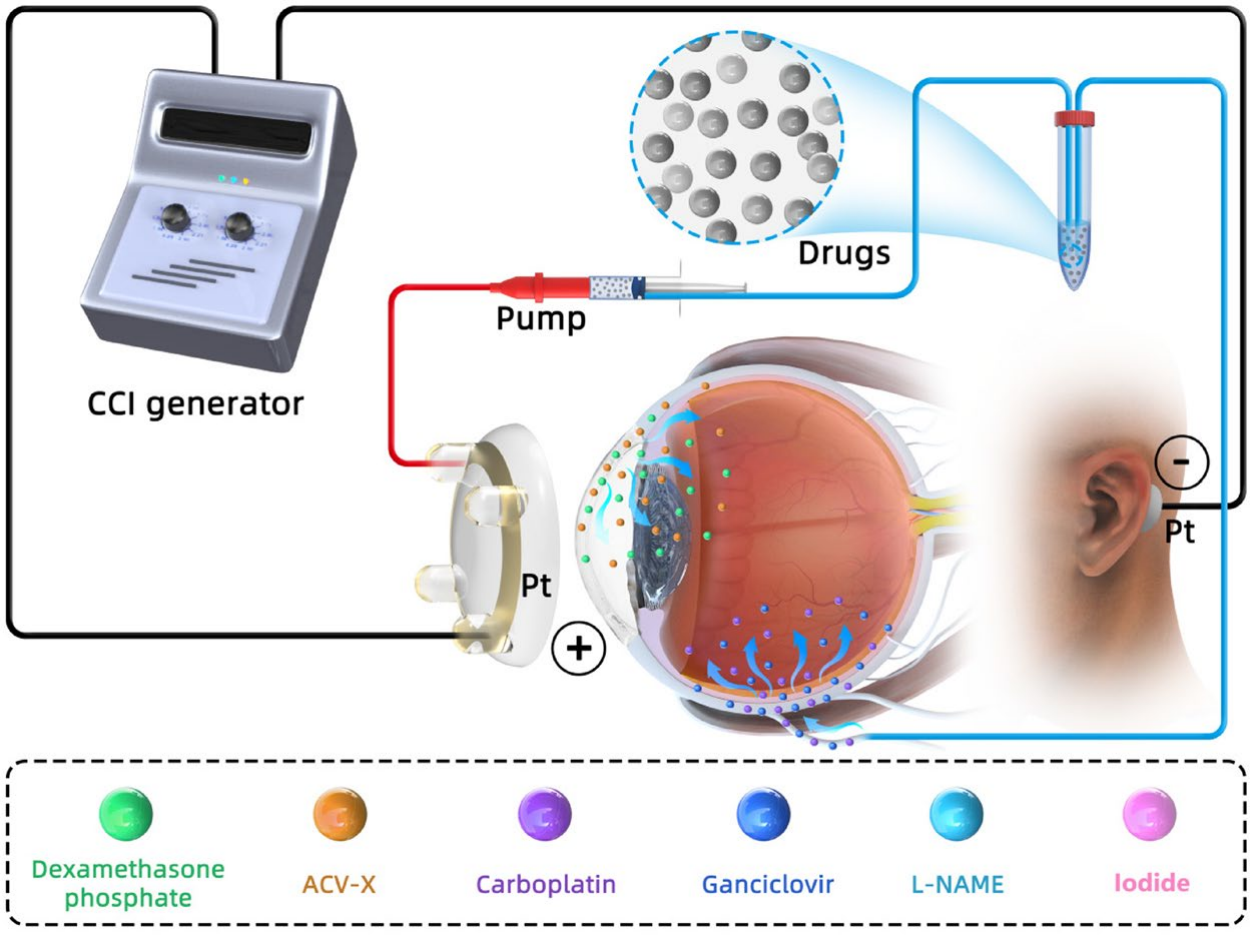
CCI delivers different drugs to against multiple ophthalmological pathologies. Abbreviations: CCI, Coulomb controlled iontophoresis; ACV-X, acyclovir (X = Arg, Gly and Trp); L-NAME, n′-nitro-L-arginine methyl ester.

Generally, iontophoresis is performed by placing the iontophoretic probe over the pars plana to generate an electric field, while another return electrode is attached to body surface. Under this condition, the pharmacological molecules serve as conducting medium of the generated current through eye tissue. The last decade has witnessed the enormous developments of iontophoresis system for efficient delivery of ophthalmic drugs. This technique has been used for transporting ophthalmic drugs, including antibiotics, steroids, peptide and gene products (Li & Hao, 2018; Perez et al., 2020). Iontophoretic devices with various capabilities have been developed for drug delivery nowadays. The most basic of iontophoretic device consists of a pair of electrodes, a power source, timers, an ampere meter, and the drug applicator (e.g. eye-cup solution or drug saturated hydrogels). For instance, OcuPhor (Iomed, Inc., Salt Lake City, Utah, USA) is an emerging custom-manufactured drug applicator that is made of polyacetal hydrogel sponge (Parkinson et al., 2003; Molokhia et al., 2020). The OcuPhor is composed of a silver-silver chloride ink conductive element, a drug saturated hydrogel pad, and a flexible wire to connect the conductive medium with the dose controller. During administration, the hydrogel matrix pad is attached to the sclera in the lower cul-de-sac of eyeball. Another return electrode is placed on the body surface to accomplish the entire electrical circuit. As a nonintrusive patented device, coulomb controlled iontophoresis (CCI; Eyegate Pharmaceuticals, Inc., USA) system (Figure 3) produces a variable electrical current across the conjunctival epithelium, resulting in electrical fields during iontophoresis (Arboleda et al., 2014). The CCI system is comprised of a generator and an iontophoresis probe. When iontophoresis was performed, the CCI ocular cup was placed on the eye and the negatively charged electrode (passive electrode) was inserted intramuscularly into the flank of the mouse to accomplish a closed circuit (Behar-Cohen et al., 1997; Hayden et al., 2006; ). Animal experiments showed that transscleral CCI for repetitive delivery of acetylsalicylic acid into the eye resulted in significant and sustained intraocular salicylic acid concentration without side effects (Kralinger et al., 2003). The CCI system can adjust automatically resistance changes to regulate the drug flow across anatomic barriers. This system uses a disposable drug-loaded hydrogel probe to efficiently deliver pharmacological molecules. In order to reduce the tissue damage caused by high current intensity, a patented device (In hydrogel ionic circuit HIC; University of Nebraska, Nebraska, USA) was invented. In this ion current-conducting circuit device, fluidic channels are filled with saturated phosphate salt solutions, and encapsulated in a polyethylene glycol (PEG) hydrogel matrix. The PEG hydrogel formed an aqueous two-phase separation with the phosphate salt solutions. This device is capable of minimizing Joule heating, effectively buffering electrochemical reaction-generated pH changes, and absorbing electrode overpotential-induced heating. As a result, this device allows safe application of high intensity currents, minimizes ocular tissue damage, and enhances intraocular macromolecule delivery (Zhao et al., 2022). The EyeGate II delivery system (EGDS; EyeGate Pharmaceuticals, Inc.) is another patented transscleral iontophoresis system to deliver ocular drugs. The EGDS has been used in multiple human clinical trials. Its safety and effectiveness has been verified in transscleral iontophoresis using dexamethasone phosphate (EGP 437; a 40 mg/mL solution designed for specific use with the EGDS) (Patane et al., 2013; Perez et al., 2020).

In the study of Sarah Molokhia et al., they did not use the commercially available iontophoresis eye applicator. Since loss of proper contact between the drug electrode and eye application site can lead to intermittent variable current density or short circuiting, the iontophoretic eye applicator like Visulex-I that fits well and has reliable contact with the surface of the eye was selected to test in vivo transscleral iontophoresis (Molokhia et al., 2020).

Hydrogels have been used as safe, comfortable, and effective probes for iontophoresis in ophthalmological practice (Gratieri et al., 2017; Jiang et al., 2021). Hydrogels are made of hydrophilic polymer with perfect water solubility. Previously, hydrogels are used for producing contact lenses and for controlled transdermal drug delivery. During iontophoresis, drug saturated hydrogels would facilitate drug handling, minimize tissue hydration, and modulating drug release kinetics (Guigui et al., 2020). Hydrogel probe can deliver a substantial amount of loaded antibiotics to the posterior segment of the eyeball. Thus far, several recent studies have used hydrogel probes to deliver therapeutic doses of antibiotics to treat ocular infections and inflammatory conditions (Eljarrat-Binstock et al., 2004; Chen & Kalia, 2018).

Since the development of iontophoresis from the twentieth century, there has been a lack of regulatory standards from device production to rational application. Moreover, many animal studies have used self-assembled devices that lack reproducibility when testing the efficacy of the technology, which may lead to large discrepancies between animal studies and clinical trials. Another issue of concern is that most of the iontophoresis techniques in animal experiments use reversible electrodes such as Ag/Agcl. On the other hand, inert electrodes such as platinum electrodes are mostly used in clinical trials. In the ionization process using inert electrodes, the PH of the anode will keep decreasing while the PH of the cathode will keep increasing. This difference in pH changes due to electrode materials may lead to enhanced toxicity or diminished efficacy of iontophoresis technique in clinical trials. Therefore, it is necessary to use a buffer to balance this PH change in clinical trials (Gratieri et al., 2017). Excitingly, more and more devices have been developed and patented for clinical use. Many clinical trials are also struggling to find a ‘gold standard’ for therapeutic use. As the scope of clinical trials expands and the safety data accumulates, a satisfied standard will eventually be established for iontophoresis.

## 4. The utilization of iontophoresis to deliver drugs against ocular diseases

### 4.1. The potential therapeutic effects against dry eye disease

Dry eye disease (DED) is one of the most common diseases of the anterior segment (Gayton, 2009). There is usually more than one factor that can cause DED. Lesions in any of the components associated with anterior segment (including the ocular surface, tear film, the interconnecting neural reflex loops, and the main lacrimal glands) can lead to the development of DED (Stern et al., 1998; Gayton, 2009). It is usually accompanied by conjunctival or corneal inflammation and increased tear film osmolarity (Anonymous, 2007). Currently, the main treatment for DED is to minimize its objective and clinical symptoms, which may improve with treatment, but cannot be cured. In addition, DED patients must use artificial tear replacement products frequently (Marshall & Roach, 2016). Therefore, a treatment that can relieve dry eye symptoms for a long time course is necessary.

Reactive oxygen species (ROS) scavengers in tears can remove ROS from the surface of the eye. However, if excessive ROS are left on the eye surface, or if tear production is reduced, ROS would impair eye tissue (Choy et al., 2000; Fujihara et al., 2000). Abundant ROS are found in the tears and conjunctival cells of DED patients, and these ROS, if not removed in time, can have toxic effects on the surface cells of the eye and aggravate the inflammatory response of the cornea or sclera (Horwath-Winter et al., 2005). Iodide is a reducing agent that has been shown experimentally to scavenge ROS (Moser et al., 1991). Clinical trials have demonstrated the safety and efficacy of iodide iontophoresis in the treatment of DED. After three months of iontophoresis treatment, the frequency of using artificial tear replacement products has been halved in patients (Horwath-Winter et al., 2005). In addition, another forward-looking, single-centered, double-masked design utilizing a controlled adverse environment (CAE) trial showed that dexamethasone phosphate, a corticosteroids anti-inflammatory agent, is effective in reducing the inflammatory response by reaching the lesion through iontophoresis, resulting in partial improvement of DED (Patane et al., 2011).

### 4.2. The potential therapeutic effects against pathologic myopia

Pathological myopia refers to other pathological changes of myopia combined with fundus, including retinal degeneration, retinal tear, retinal detachment, macular hemorrhage and vitreous opacity. Scleral localized ectasia, the typical characteristic of eye deformation in myopia, is often associated with scleral thinning and weakening (Rong et al., 2017). In an therapeutic trial against animal myopia, Rong et al. modified the scleral riboflavin/ultraviolet A (UVA) cross-linking procedure with an iontophoresis-assisted drug delivery system. The abnormal elongation of the myopic eye was effectively controlled 1 month after the treatment and even almost halted 3 months after the treatment. The result suggests that the alteration in collagen metabolism may be triggered off by the treatment, and the modified scleral cross-linking procedure may be a potential method to control the pathologic process of myopia. Iontophoresis has also been used in clinical studies to enhance the penetration of riboflavin. Recent clinical studies have found that iontophoresis-assisted corneal cross-linking (I-CXL) is less effective than conventional cross-linking (C-CXL) in terms of the keratometry measures and cross-linking extent as indicated by the demarcation line. However, I-CXL is associated with faster improvement in visual acuity and less epithelial damage/corneal swelling (Perez et al., 2020).

### 4.3. The potential therapeutic effects against uveitis

Most of the multiple pathological changes in the eye are associated with uveitis. Although it accounts for a small percentage of global visual impairment, approximately one-third of patients with uveitis will meet the criteria for legal blindness (Muñoz-Fernández & Martín-Mola, 2006; de Smet et al., 2011; Miserocchi et al., 2013). Uveitis can be divided into anterior segment and posterior segment subtypes, depending on the segment in which it occurs. Inflammation of the anterior segment mainly accumulates in the anterior chamber, conjunctiva, ciliary body and surrounding tissues. Inflammation in the posterior segment mainly accumulates in the retina and its surrounding blood vessels (Ruiz-Moreno et al., 1992).

For the treatment of inflammatory diseases, a relatively wide range of therapeutic drugs, such as immune agents, antibiotics, non-steroidal drugs and corticosteroids have been applied (Myles et al., 2005). In therapeutic experiments of endotoxin-induced-uveitis (EIU) in rats model, corticosteroids such as dexamethasone have been shown to achieve therapeutic concentrations in the anterior and posterior segments using iontophoresis techniques (Behar-Cohen et al., 1997). In addition, anti-sense oligonucleotides (ODNs) also have the potential capacity to treat against inflammatory conditions (Glover et al., 1997). Nitric oxide synthase (NOSII) is a key enzyme specific to EIU and therefore its decreased gene expression has potential benefits for the uveitis. However, ODNs cannot cross the biological barriers and reach the posterior segment. It was demonstrated that in the EIU rat model, ODNs did reach therapeutic concentrations in the anterior and posterior segments by iontophoresis (Voigt, de Kozak, et al., 2002). Meanwhile, there is a more direct approach in which the introduction of the NOSII inhibitor N'-nitro-L-arginine methyl ester (L-NAME) into the uvea of EIU rat using iontophoresis can partly ameliorate the inflammatory response (Behar-Cohen et al., 1998). Furthermore, recent clinical trials of noninfectious anterior uveitis treated with dexamethasone have consistently shown effective improvement of therapeutic efficacy using noninvasive iontophoresis. These clinical trials use anterior chamber cell (ACC) scores to evaluate the efficacy of iontophoresis treatment. The researchers ultimately conclude that treatment with low doses of dexamethasone by iontophoresis is effective and well-tolerated. They propose that dexamethasone treatment using iontophoresis may have better efficacy in severe noninfectious anterior uveitis (Cohen et al., 2012; Sheppard et al., 2020).

### 4.4. The potential therapeutic effects the herpes simplex infections

Herpes simplex is a skin viral disease in which herpes simplex virus HSV infects the skin and mucosa. Clinically, herpes simplex virus is divided into two types, herpes simplex virus type 1 and herpes simplex virus type 2. Herpes simplex virus (HSV), particularly HSV-1 can affect various components of the human eye, causing herpes simplex keratitis, uveitis, retinitis or endophthalmitis (Chen & Kalia, 2018).

Previous researchers have investigate the transcorneal and transscleral iontophoresis of biolabile amino acid ester prodrugs of aciclovir (ACV-X, X = Arg, Gly and Trp). They found that although the high aqueous solubility of ACV-X prodrugs enhanced passive transcorneal delivery of ACV species, short-duration iontophoresis resulted in significantly greater transcorneal permeation and corneal deposition of ACV species using less concentrated prodrug solutions. As evidenced by the ACV-Gly results, the drug permeation through sclera by iontophoresis showed a significant increase compared with passive diffusion. Due to the high intrinsic permeability of the sclera, the penetration rate of transscleral iontophoresis is much greater than that of transcorneal iontophoresis. Biodistribution studies further confirmed the superiority of short-duration iontophoresis, as the average concentration of ACV species in the eye globes following iontophoresis of ACV-Gly for five minutes was greater than the IC_50_ of ACV against HSV-1 (Chen & Kalia, 2018). In summary, iontophoresis of these water-soluble ionizable ACV-X prodrugs may prove to be useful for the treatment of HSV infections in both the anterior and posterior segments (Chen & Kalia, 2018). Intriguingly, the iontophoresis of adrenaline and Timolol (Hill et al., 1987) can induce the recurrent infection of ocular herpes. The shedding frequency induced by epinephrine iontophoresis is the highest among all methods. Kwon et al. (1981) confirmed that epinephrine iontophoresis induced ocular HSV-1 shedding reliably and with a high frequency in the latently infected rabbits. They therefore proposed that viral shedding offers a system for studying the factors involved in recurrent HSV-1 ocular infections.

### 4.5. The potential therapeutic effects against noninfectious scleritis

Noninfectious scleritis is a rare eye disease that is accompanied by redness and severe ocular pain (Murthy et al., 2020; Nevares et al., 2020). Corticosteroid, nonsteroidal anti-inflammatory and topical immunosuppressives are considered as the first-line drug for noninfectious scleritis except for limitations in efficacy and side effect (Dutta Majumder et al., 2020; Abdel-Aty & Kombo, 2021). Perhaps the iontophoresis system can accurately deliver drugs to the eye and reduce the occurrence of side effects. Iontophoresis of lithium was employed for the treatment of scleritis as early as 1927 (Sarraf & Lee, 1994). In recent years, this idea has been further confirmed by a series of studies. A randomized, double-masked, dose-escalating study of iontophoretic delivery of EGP-437 with mild to moderate noninfectious, non-necrotizing anterior scleritis was performed. It was effective in the lowest electric field dose, and 5/7 eyes achieved the preliminary effect within 28 days. In the middle dose group, 0/7 eyes within 7 days, 1/7 eyes within 14 days, and 2/7 eyes within 28 days met the primary outcome. In the highest dose group, 2/6 eyes within 7 days met the primary outcome. Obviously, A lower electrical field dose appears the most promising, perhaps because the site of inflammation is superficial and the higher electric field doses drive the medication too deep, surpassing the level of inflammation. The researchers propose that the iontophoretic treatment is well-tolerated and safe for the treatment of scleritis (O’Neil et al., 2018).

### 4.6. The potential therapeutic effects against diabetic retinopathy

Diabetes is a endocrinological disease worldwide, and its most common ocular complications, such as diabetic retinopathy (DR), are therefore of increasing concern (Cheung et al., 2010). It is estimated by the International Diabetes Federation that as many as 463 million people worldwide will be affected by diabetes in 2019, with DR accounting for 22.27% (approximately 103.12 million) of the total number of people with diabetes (Saeedi et al., 2019; Teo et al., 2021). Moreover, DR is closely associated with blindness in both prime and elderly populations (Barber, 2003; Cheung et al., 2010).

The pathogenesis of DR is a series of reaction cascade secondary to the endothelial cell damage that caused by prolonged hyperglycemia in the retinal vasculature. For example, hyperglycemia-induced oxidative stress, inflammatory response, protein kinase C activation, and upregulation of vascular endothelial growth factor (VEGF) lead to retinal vascular value-added lesions, vitreous hemorrhage, retinitis, and even retinal detachment (Dong et al., 2009; Cheung et al., 2010). Although many etiological factors are involved, the dominance of VGEF especially in proliferative DR is undisputed (Wirostko et al., 2008).

Therefore, anything that lowers VEGF or addresses the underlying cause of elevated VEGF (hyperglycemia) can improve DR therapy. Bevacizumab is a humanized IGg1 antibody that binds primarily to VEGF with high affinity to reduce VEGF. But it cannot passively pass through the sclera to reach therapeutic concentrations in the posterior segment. Thus a new study attempted to use iontophoresis to overcome the inability of bevacizumab to pass through the sclera. They concluded that anodic iontophoresis could increase the amount of bevacizumab passing through the sclera, thus demonstrating the promising use of iontophoresis for DR (Pescina et al., 2010). It has been shown that insulin can indeed be introduced into the posterior through the transscleral iontophoresis and achieve a therapeutic effect by lowering blood glucose (Myles et al., 2005).

### 4.7. The potential therapeutic effects against retinoblastoma

Retinoblastoma (Rb) is the most common primary intraocular malignancy of childhood, and approximately 9000 new cases occur worldwide each year (Ortiz & Dunkel, 2016). Rb, which originates from the retina, is a malignancy associated with somatic mutation or germline mutation (Rao & Honavar, 2017). Although systemic chemotherapy is one of the main treatments for Rb, it can still lead to secondary tumors in populations with RB1 mutation. Local chemotherapy can provide therapeutic or palliative benefits at all stages of Rb, while protecting patients from the toxic and side effects of systemic administration (Byrne et al., 2018). As a DNA alkylating agent, carboplatin, is commonly administered for treating Rb with minimal side effects (Narayana et al., 2021). The transscleral CCI of carboplatin has been tested in mouse models of retinoblastoma (Hayden et al., 2006). The mice were divided into groups with different carboplatin concentrations and received iontophoresis for 5 minutes. CCI delivery of carboplatin safely and effectively controls intraocular tumors in a dose-dependent manner. In particular, at carboplatin concentrations of the maximum solubility in balanced saline solution (BSS) all treated eyes (9/9, 100%) exhibited complete tumor control. The safety and efficacy of focal carboplatin delivery has also been verified by another experiment (Hayden et al., 2004). Rabbit eyes were treated with different conditions, including a single intravenous infusion of carboplatin, and a single application of carboplatin delivered by CCI. After iontophoretic carboplatin delivery, the concentration in retina, choroid, vitreous humor and optic nerve was significantly higher than that of intravenous administration. Iontophoretic focal delivery of carboplatin may deliver high-dose chemotherapy to the vitreous and posterior segments without the risks associated with globe penetration. Moreover, no toxicity in rabbit eyes after transscleral CCI applications of carboplatin was determined (Hayden et al., 2004), suggesting that the local iontophoresis may act as an auxiliary means for the treatment of Rb.

### 4.8. The potential therapeutic effects against cytomegalovirus retinitis

Cytomegalovirus retinitis (CMVR) is a disease of immunocompromised host, often associated with severe vision loss due to retinal atrophy, retinal detachment and/or optic nerve atrophy (Port et al., 2017; Suzuki et al., 2021). A patient with CMVR received intravitreal injection of foscarnet and oral valganciclovir for 2 months, and his CMVR retinitis was significantly improved (Chaudhry & Fung, 2021). Perhaps combining the therapeutic drugs of CMVR with iontophoresis is a promising strategy. In order to study the feasibility of delivering ganciclovir into the vitreous/retina with transscleral iontophoresis, Tim et al. measured the levels of ganciclovir in the vitreous/retina several times after a single application of transscleral iontophoresis in rabbits. They found there was still detectable level of ganciclovir in the vitreous/retina even 72 hours later. Therefore, ganciclovir can be successfully delivered into the vitreous for treatment of CMVR (Lam et al., 1994). Another study has also proved the effectiveness of iontophoresis in the treatment of CMVR. Marc et al. found that ocular iontophoresis can increase the concentration of foscarnet in vitreous obtained by intravenous injection (Yoshizumi et al., 1996). They propose that ocular iontophoresis may be an important and noninvasive local treatment technology for these patients who need intermittent ocular drug supplement and cannot tolerate higher dose of systemic antiviral drugs (Yoshizumi et al., 1996). Therefore, iontophoresis may be a good assistance scheme for local management of CMVR.

### 4.9. The potential therapeutic effects against choroidal neovascularization (CNV)

Choroidal neovascularization (CNV) is secondary to many fundus diseases, most commonly seen in wet age-related macular degeneration (mostly in the middle-aged and elderly, the older the age, the higher the incidence rate) and pathological myopia (mostly in high myopia (> U0 degrees). In addition, CNV may be complicated by fundus angioid stripes, hereditary macular degeneration, inflammation (central exudative chorioretinitis, etc.), trauma, tumor or no obvious cause (idiopathic choroidal neovascularization).

By 2010, only two anti-angiogenic drugs have received Food and Drug Administration (FDA) and European Medicines Agency approval for the treatment of neovascular AMD, namely Ozurdex (dexamethasone) and Pegaptanib (Anderson et al., 2010). In addition, both bevacizumab and ranibizumab are anti angiogenic drugs. In 2011, S. Kevin Li, studied the effective electrophoretic mobilities and charges to provide information for the development of drug ionic conductance. The results show that bevacizumab and ranibizumab have low electrophoretic mobilities (Li et al., 2011). When the time comes to 2020, Sarah Molokhia et al. studied the importance of electroosmosis on macromolecules of low charge to mass ratio, and to evaluate transscleral iontophoresis efficacy in a choroidal neovascularization (CNV) animal model. They found that Galbumin, a large molecule with significant negative charge (−27), can be delivered into the eye through anodal iontophoresis (Molokhia et al., 2020). According to their drug distribution data, penetration with similar molecular weight molecules does reach the retina/choroid layers. Transscleral iontophoresis of IgG at 4 mA with a low ionic strength formulation was about 600 times greater than passive diffusion and 14-fold over a conventional formulation in vitro. Approximately 0.6 mg of bevacizumab can be delivered into the rabbit eye in vivo with a 20-min treatment of iontophoresis (Molokhia et al., 2020). In the CNV model, the iontophoresis treatment delayed retinal neovascularization by 4 weeks. Therefore, researchers put forward that the effect of altering ion competition in iontophoretic transport by the low ionic strength formulation could contribute to the enhanced delivery.

## 5. Factors affecting iontophoresis

Several factors can affect the efficiency of iontophoresis, such as the current density, duration of stimulation, concentration of drug, pH value and permeability of tissue (Hughes & Maurice, 1984). First of all, in iontophoresis, the current density and duration of stimulation are closely related to the total amount of drugs. The current density is proportional to the electric field intensity.

The electric field force on the drug ions would increase in parallel with the augment of current density. Thus more ions are delivered within a fixed period of time.

Moreover, the longer the stimulation time is, the more drug ions would be delivered. Although iontophoresis has the advantage of enhancing the bioavailability of drugs, we should pay attention to the toxic damage that caused by high current density and the long length of iontophoretic duration (Gratieri et al., 2017). Clinical trials have verified that an electric current with the density of 20 mA/cm^2^ for 5 min would be harmless to the corneal tissue (Voigt, Kralinger, et al., 2002; Hao et al., 2009; Patane et al., 2011). Previous study also shows that an electric current with the density of 5.0 mA/cm^2^for 10 min would not cause any intraocular complications in transscleral iontophoresis (Rieger et al., 2010; Jung et al., 2018). However, the higher electric current (with the densities of 100–Y0 mA/cm^2^) would cause retina and choroid burns, hemorrhagic necrosis, edema and infiltrations in the subjects (Yoshizumi et al., 1995; Eljarrat-Binstock et al., 2010; Rahić et al., 2020). These unwanted damages may be closely correlated with the area of application site, the length of iontophoretic duration, and the intensity of applied current. Additionally, if the concentration of drug is small, drug concentration and tissue permeability are positively correlated with the amount of drug ($m_{d}$) passing through the epithelium. This may be expressed as the following:

$$m_{d}=\frac{\;iP_{d}C_{d}t}{F({{{P_{i}C}_{i}+P}_{d}C}_{d})}$$

$$m_{d}=\frac{\text{ i*t}}{F(1+\frac{{P_{i}C}_{i}}{P_{d}C_{d}})}$$

where $P_{d}$ and $C_{d}$ represent permeability and concentration of the drug, and the duration of iontophoresis is represented by t. F is Faraday’s constant, and ${P_{i}C}_{i}$ is the sum of the products of the Penetration rate and concentrations of any small monovalent ions that carry the current besides drugs. The actual amount of drug penetrating the epithelium is $m_{d}$ and i represents the per unit area of current (Hughes & Maurice, 1984). Even though this relationship comes from the experimental conclusion of corneal pathway, it still has guiding significance for scleral pathway. Moreover, it should be noted that $P_{d}C_{d}$ cannot be much larger than ${P_{i}C}_{i}$, otherwise $m_{d}$ will not change with the change of $P_{d}C_{d}$. In iontophoresis, the pH of the cathode may show an upward trend and the pH of the anode may show a downward trend, but the pH change may not be particularly obvious during the gradual penetration of drug ions. However, the change of pH does affect the maximum ionization value and mobility of ions (Guffey et al., 1999; Eljarrat-Binstock & Domb, 2006).

The variability of the permeability coefficient data of the macromolecules in transscleral transport can be partly attributed to the differences in the thicknesses among human, porcine, bovine, and rabbit sclera (Pescina et al., 2015).The permeability coefficients of sclera between human and other animal species are listed as the following order: rabbit > human > porcine > bovine (Li & Hao, 2018). However, even after taking the variation of scleral thickness into consideration, significant data variability is still observed within and between the animal species In general, the permeability coefficient decreases when the molecular weight of the permeant increases. However, Li and Hao found the difference between the experimental and theoretical slope values, uncovering a steep-size dependence of the diffusion relationship (Li & Hao, 2018). They proposed that it could be attributed to the hindered diffusion across the sclera (Chopra et al., 2010; Molokhia et al., 2020). They also pointed that the variability is not limited to macromolecules and can be observed over the whole molecular weight range for the molecules (Li & Hao, 2018). In general, there is a trend of smaller variability from permeants of higher permeabilities. The causes of the variability in these studies are not known and can be related to a number of factors including the experimental conditions: e.g. diffusion apparatus (diffusion cell) setup, temperature, solution (or formulation) containing the permeant, and duration of the permeation studies (Li & Hao, 2018). Quantitative structure permeation relationships (QSPR) for electroosmosis suggests an improvement in the effect of electroosmosis with increasing permeant molecular weight. In addition, by adjusting the magnitude of electric field, a substantial amount of pharmacological molecules could be transported precisely to target tissue. Notably, the contact area of the antibiotics probe with the ocular surface is also critical, since it would affect the intensity of the applied current.

## 6. Toxic effects of iontophoresis

Iontophoresis has been used in many clinical trials to treat eye diseases. However, this procedure can produce potential complications, including epithelial edema, decrease in endothelial cells, inflammatory infiltration and burns. These damages are reliant upon the site of application, the current density and the iontophoretic duration (Myles et al., 2005).

The toxicity of transscleral iontophoresis to the eyeball is closely correlated with the applied current density. High current density for iontophoresis, may cause choroidal and retinal damages. Lam et al. (1991) use 531 mA/cm^2^ current density for scleral iontophoresis, and find that the size and severity of rabbit retinochoroidopathy increase with the extension of application time. As 1 min after iontophoresis, no retinopathy is found by light microscopy. However, Ion osmotic therapy for 5 and 15 min results in necrosis of retinal pigment epithelium (RPE), proliferation of macrophages, and loss of retinal nuclear layer. On the other hand, another study shows that after several weeks of gentamicin iontophoresis through sclera with extremely high current density (765.3 mA/cm^2^) (Barza et al., 1986) for 10 minutes, the monkey electroretinogram is normal. The discrepancy in safety results shows that the toxicity of large current to the eyeball retina is not stable, which may be related to the iontophoretic duration, the type of imported drugs and the current density, or the protocol difference of toxicity test results. Behar Cohen et al., have reported a safety procedure for transscleral iontophoresis with current intensity less than 50 mA/cm^2^ (Behar-Cohen et al., 2002). Within this safety procedure, many studies have not found any evidence of inflammation or toxicity to the intraocular tissues during iontophoresis treatment for at least one week.

Several researches show that after corneal iontophoresis with different current densities and drug types (dexamethasone phosphate, tobramycin, etc.), corneal epithelial cell edema and endothelial cell count decline occurred within a short period of time (1–10 min) (Rootman et al., 1988; Patane et al., 2013). If the current density of 0.8 mA/cm^2^ is used for iontophoresis for 5 min, small surface epithelial abnormalities would occur immediately in the cornea, and local epithelial edema would occur immediately 10 minutes later. When the current density of 0.8 mA/cm^2^ is used for vancomycin iontophoresis for 5 minutes, the endothelial cell count would decrease by 8.8% and 5.4%, respectively. In addition, the toxicity of corneal opacification (Grossman & Lee, 1989) and interstitial edema (Eljarrat-Binstock et al., 2004) are also reported when iontophoresis are introduced through the cornea.

## 7. Conclusion

In summary, the complicate architecture of eyeball induces several special challenges during drug delivery. Effective delivery techniques for will be necessary for drug application, with preference for noninvasive modes. The main aim of future advancements in this zone is to enhance the bioavailability of drug, prolong the pharmacological activities, minimize toxic reactions and achieve patient compliance. Recent developments in the iontophoresis equipment and the biopharmaceutical probes would collectively lead to the extensive clinical use of this technique in ophthalmological practice. The key advantage of ocular iontophoresis is its noninvasive application which might enable satisfied patient compliance. The feasibility of using iontophoresis for ocular drug delivery has been demonstrated in a series of animal models and clinical trials. The development of iontophoresis technology is well established in terms of devices and mechanisms, and it is foreseeable that this noninvasive method of drug delivery will certainly be the mainstay of research and development for ophthalmic drug administration in the future. Further developments of iontophoresis equipments, drug probes, and smart accessories are necessary to build up optimal protocols for clinical use. One of the main challenges of iontophoresis is to develop clinically available and low-cost devices to provide efficient transocular drug delivery. There are few devices approved by FDA for ion introduction into the eye, most of which are used to replace hypodermic injection or introduce drugs into the tympanic membrane. In addition, the efficiency of iontophoresis can also be improved by gradual uncovering the principle of iontophoretic transport. For example, ion competition in iontophoretic transport can be altered by the low ionic strength formulation, which could contribute to the enhanced delivery. The main problem facing this technology is the lack of clinical practice and the consequent problem that it requires a harmless and therapeutic criterion (correct voltage, current and duration). The lack of funding to promote this technology to medical institutions is currently another reason that preventing this technology from entering the clinic practice. More attention should be paid to this promising technology to enhance its future clinical utilization.
